# Association Between Physical Function, Pulmonary Function, and Social Determinants of Health in Individuals with Post-Tuberculosis Lung Disease

**DOI:** 10.3390/arm94040052

**Published:** 2026-07-27

**Authors:** Nielza Moreira de Souza, Pedro Henrique Perpetuo de Lima Silva, Estephane Ramos de Souza Penna, Amanda Oliveira dos Anjos, Alícia Sales Carneiro, Walter Costa, Bruna Cuoco Provenzano, Ana Paula Santos, Agnaldo José Lopes

**Affiliations:** 1 Rehabilitation Sciences Post-Graduation Program, Augusto Motta University Center (UNISUAM), Rua Dona Isabel, 94, Bonsucesso, Rio de Janeiro 21032-060, Brazil; nielzasouza@souunisuam.com.br (N.M.d.S.); pedroperpetuo@souunisuam.com.br (P.H.P.d.L.S.); 2 Faculty of Physiotherapy, Augusto Motta University Center (UNISUAM), Av. Paris, 84, Bonsucesso, Rio de Janeiro 21041-020, Brazil; estephanepenn@gmail.com (E.R.d.S.P.); amandaxdosanjos@gmail.com (A.O.d.A.); 3 Department of Pulmonology, Pedro Ernesto University Hospital, Universidade do Estado do Rio de Janeiro (UERJ), Boulevard 28 de Setembro, 77, Vila Isabel, Rio de Janeiro 20551-030, Brazil; aliciasc6@gmail.com (A.S.C.); dr.waltercosta@terra.com.br (W.C.); brunaclinicamedica@gmail.com (B.C.P.); anapsantos.ip@gmail.com (A.P.S.); 4 Postgraduate Program in Medical Sciences, School of Medical Sciences, Universidade do Estado do Rio de Janeiro (UERJ), Avenida Professor Manoel de Abreu, 444, 2º Andar, Vila Isabel, Rio de Janeiro 20550-170, Brazil; 5 Medical School and Institute of Thorax Diseases, Universidade Federal do Rio de Janeiro (UFRJ), Rua Prof. Rodolpho Paulo Rocco, 255, Sala 01D 58/60, Cidade Universitária, Rio de Janeiro 21941-913, Brazil

**Keywords:** tuberculosis, exercise, respiratory function tests, muscle strength

## Abstract

**Highlights:**

**What are the main findings?**

- Individuals with post-tuberculosis lung disease (iwPTLD) have low functional exercise capacity, impaired lung function, and deteriorating social determinants of health (SDoH), even when they have adequate housing.
- Although not negligible, the association between physical function, pulmonary function, and SDoH is weak to moderate in this patient population.

**What are the implications of the main findings?**

- When monitoring iwPTLD, it is important to consider not only physiological parameters, but also socioeconomic and environmental factors that contribute to overall functional limitation.
- These findings may serve as a basis for randomized controlled trials applying social protection measures during and after tuberculosis treatment, in order to better clarify the impact of socioeconomic conditions on physical performance.

**Abstract:**

Although poverty and tuberculosis are insidiously linked, knowledge of the relationship between social determinants of health (SDoHs) and post-tuberculosis lung disease (PTLD) is limited. This study aimed to analyze the association between physical function, pulmonary function, and SDoHs in individuals with PTLD (iwPTLD), considering the impact of social inequalities on physical performance. This cross-sectional study collected social data from 69 iwPTLDs using a standardized assessment form. The patients underwent pulmonary function testing via spirometry and body plethysmography, as well as respiratory muscle strength and quadriceps muscle strength (QMS) testing. They also completed the six-minute step test (6MST). The median value of steps climbed by participants on the 6MST was 88 (57–117), corresponding to 50.1% (34.9–73.2) of the predicted value. The mean QMS was 28.7 ± 11.9 kgf, with 11 participants (17.4%) showing QMS below the cutoff point. Spirometry revealed normal, obstructive, restrictive, and mixed patterns in 19 (27.5%), 20 (29%), 18 (26.1%), and 12 (17.4%) of the participants, respectively. Performance on the 6MST showed no statistically significant association with SDoHs. QMS showed a statistically significant association with treated sewage (*W* = 84, *p* = 0.026). Forced expiratory volume in one second showed significant correlations with education level (*ρ* = 0.248, *p* = 0.040), social protection (*W* = 207, *p* = 0.050, *r* = 0.238), and treated water (*W* = 24.5, *p* = 0.029, *r* = 0.264). Maximum inspiratory pressure showed significant correlations with education level (*ρ* = 0.246, *p* = 0.042) and treated water (*W* = 20, *p* = 0.021, *r* = 0.280). The regression model for 6MST and QMS performance showed that 12% and 45% of the variability was explained by the studied variables, respectively. In iwPTLD, impairments in physical function and damage to lung function are weakly associated with the deterioration of SDoHs. While this relationship is weak, it should not be ignored because it may operate through indirect pathways.

## 1. Introduction

Post-tuberculosis lung disease (PTLD) is one of the most concerning lung conditions worldwide today due to its significant impact, high prevalence, and associated morbidity and mortality [1]. Up to 50% of tuberculosis (TB) survivors have sequelae, which increase their risk of death sixfold compared to individuals without the disease [1,2]. PTLD has a significant impact, accounting for 47% of the total 122 million disability-adjusted life years related to TB in new cases [3]. The most common residual lung lesions are pulmonary fibrosis, bronchiectasis, parenchymal destruction, and cavitations. These lesions reduce pulmonary compliance and compromise regional ventilation. Depending on the extent and location of the lesions, the functional pattern on spirometry can vary between obstructive, restrictive, or mixed [4]. These alterations directly impact functionality, making it difficult to perform activities of daily living (ADLs).

Frequent spirometric abnormalities occur after completion of pulmonary TB (PTB) treatment, with obstructive, restrictive, or mixed patterns being common. A significant reduction in pulmonary diffusion that is inconsistent with spirometry results may indicate pulmonary hypertension or structural lung disease [5]. However, PTLD is not merely residual lung disease; many individuals have physical deconditioning and musculoskeletal changes resulting from the prolonged period of illness and inactivity. There is a significant reduction in lean mass, primarily in the peripheral muscles of the lower limbs, due to persistent systemic inflammation and prolonged physical inactivity [6]. Individuals with PTLD (iwPTLD) often have ventilatory limitations and exercise intolerance, reflecting a long-term inflammatory and destructive process in lung tissues [1]. In assessing functional exercise capacity (FEC) in chronic respiratory diseases (CRDs), the six-minute step test (6MST) is a simple, safe, low-cost field test that shows good correlation with other functional tests, such as the six-minute walk test [7].

The World Health Organization defines social determinants of health (SDoHs) as, “non-medical factors that influence health outcomes. the conditions in which people are born, grow, work, live, and age, and the wider set of forces and systems shaping the conditions of daily life” [8]. These encompass social and economic policies, legal systems and social and group norms, which determine the global distribution of money, power and resources, as well as the conditions that influence a wide range of health-related risks and outcomes [9]. Historically, TB has been considered a classic example of a ‘social disease’, with its occurrence intrinsically linked to SDoHs. Controlling TB requires social, economic, and environmental interventions that address factors such as poverty, malnutrition, alcohol abuse, poor housing conditions, indoor air pollution, unemployment, and low educational attainment [10,11]. The extent to which TB-related SDoHs influence PTLD remains unclear [2,3].

Although SDoHs has not yet been explored in the PTLD, the social stigma associated with TB remains a significant barrier to social reintegration: iwPTLD report experiencing discrimination, shame, and isolation. This social isolation can lead to increased psychological stress, which may exacerbate physical symptoms such as dyspnea and fatigue. These symptoms can negatively impact functional capacity. Furthermore, PTLD can affect work capacity and generate economic hardship, creating a cycle of social and psychological vulnerability [12]. Post-treatment costs are estimated to be almost 15 times higher than pre-treatment costs due to indirect expenses and loss of monthly income [13]. Even after being cured, significant functional impairment remains, as evidenced by reduced aerobic capacity, increased perceived exertion, and changes in body composition. These factors can negatively impact the socioeconomic conditions of iwPTLD [14]. A complex interaction between the host, the pathogen, and the environment is believed to underlie the development of PTLD [15].

The structural and functional consequences of PTLD can impair physical performance and exercise tolerance. Beyond physiological aspects, socioeconomic and environmental conditions can potentially play a significant role in the functional recovery of these individuals by influencing their access to healthcare and their engagement in rehabilitation activities. Furthermore, prolonged functional limitation increases dependence on medical care, reduces productivity, and raises personal and family costs. Thus, functional and physical losses can hinder the return to work and ADLs, resulting in financial losses for iwPTLD [13]. Advanced age, smoking, HIV infection, late diagnosis, and precarious socioeconomic conditions are risk factors for PTLD; however, evidence regarding the social impact of PTB after microbiological cure is limited [5]. Understanding how SDoHs influence iwPTLD functionality may enable the development of rehabilitation strategies that promote autonomy, functionality, and quality of life (QoL). This study aimed to analyze the association between physical function, pulmonary function, and SDoHs in iwPTLD, considering the impact of social inequalities on physical performance.

## 2. Materials and Methods

### 2.1. Ethical Issues

The study was conducted in accordance with the Declaration of Helsinki and was approved by the Research Ethics Committee of the Pedro Ernesto University Hospital at the State University of Rio de Janeiro (protocol number CAAE-70493823.5.0000.5259, approved 25 August 2023). Informed consent was obtained from all participants.

### 2.2. Study Design

From August 2025 to April 2026, a cross-sectional study was conducted with iwPTLD at the Pedro Ernesto University Hospital at the State University of Rio de Janeiro (HUPE-UERJ) in Rio de Janeiro, Brazil. The inclusion criteria were iwPTLD aged 18 years or older who had completed anti-PTB treatment up to two years prior. The following exclusion criteria were applied: diagnosis of a neurological or musculoskeletal disease that would limit performance of the proposed tests; presence of decompensated heart disease or hemodynamic instability; presence of cognitive limitations that would prevent understanding of the instructions; and inability to perform the physical tests due to intense pain or extreme fatigue.

### 2.3. Instruments and Measurements

Social data were assessed using a standardized evaluation form based on the SDoHs previously described for PTB [10,16,17]. Thus, the collected variables included data on ethnic-racial aspects, family composition, socioeconomic conditions (monthly income, household economic participation, and receipt of social protection), housing conditions (regular garbage collection, treated sewage, and treated water), health conditions (hypertension and diabetes mellitus), and lifestyle habits (smoking and physical activity).

Pulmonary function tests, consisting of spirometry, body plethysmography, and respiratory muscle strength measurement, were performed using an HDpft 3000 device (nSpire Health, Inc., Longmont, CO, USA). Brazilian predicted values were adopted for comparison with the participants’ absolute values [18,19,20]. Obstructive and restrictive patterns were defined as forced expiratory volume in one second (FEV_1_) divided by forced vital capacity (FVC) and total lung capacity (TLC) below the lower limits of normal, respectively [21]. Brazilian predicted values were used for comparison with the participants’ absolute values [7].

Quadriceps muscle strength (QMS) was assessed using a tensile dynamometer (model E-lastic 5.0, E-sporte SE, Aracaju, Brazil). This measurement was taken after a 5 s contraction of the dominant leg and the highest value among three attempts, with 1 min intervals between each attempt, was selected for analysis. The cutoff points used were 25.3 kgf for men and 14.8 kgf for women [22].

**Figure 1:**
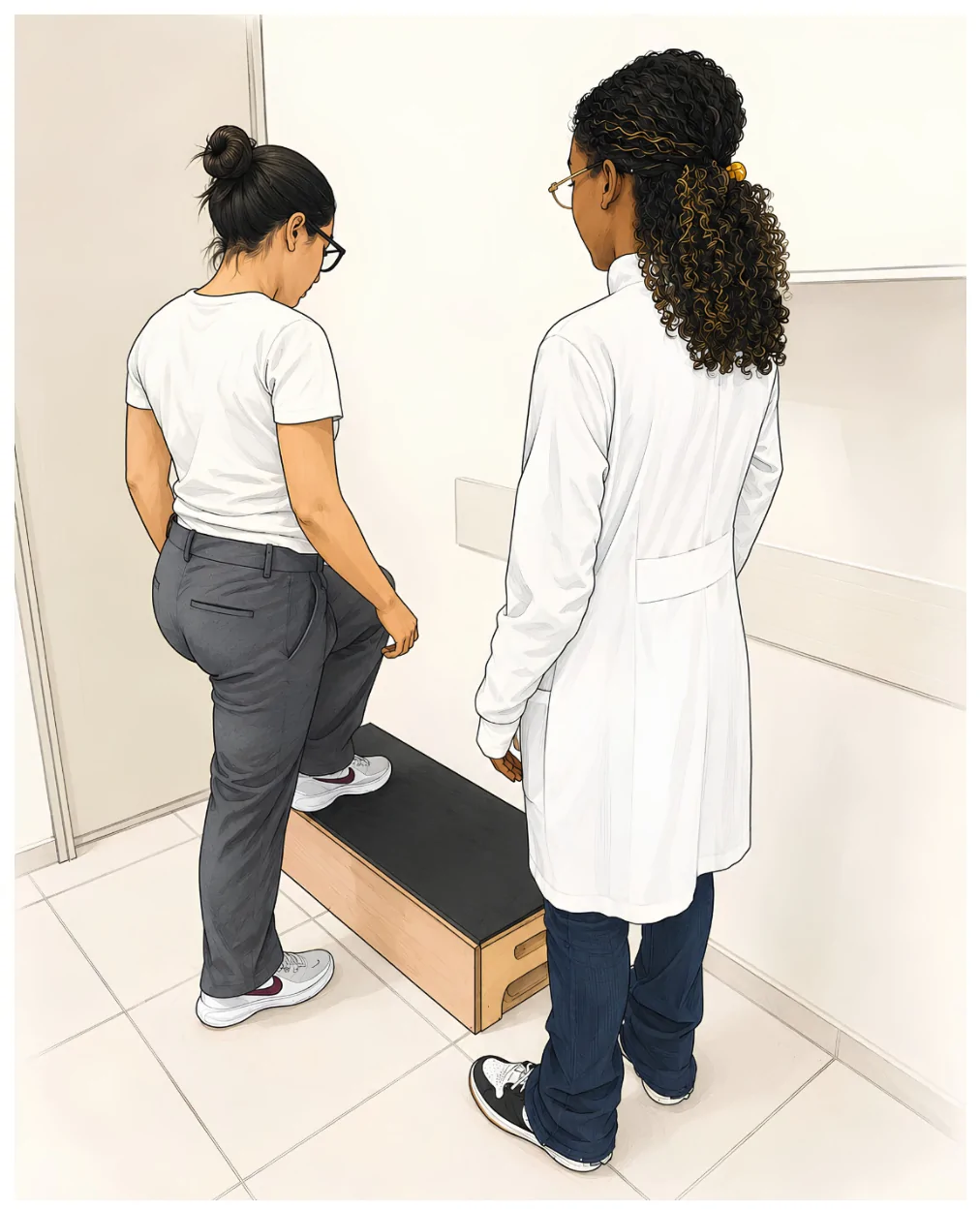
Illustration demonstrating the six-minute step test. Participants received standardized instructions at the beginning of the test and were then told to step up and down on the step as many times as possible for six minutes.

FEC was assessed using the 6MST with a 20 cm-high, 40 cm-wide, 60 cm-long wooden step without armrests (Figure 1). The 6MST was interrupted if participants experienced intolerable dyspnea, chest pain, dizziness, leg cramps, or oxygen desaturation below 85% [7].

### 2.4. Statistics

The sample size was calculated using MedCalc^®^ version 8.2 (MedCalc Software, Mariakerke, Belgium) software. The calculation took into account a moderate effect size of 0.5, a 5% significance level, and an 80% statistical power to minimize the risk of type I and type II errors. A minimum of 64 participants was required to detect differences or associations between functionality and social aspects. The study’s main outcome was the association between 6MST performance and monthly income.

Statistical analyses were conducted in the R environment (R Core Team, 2025). The normality of the data distribution was verified using the Kolmogorov–Smirnov test. Due to the ordinal nature of some of the predictor variables and the absence of a normality assumption, associations between physical function variables and SDoHs were investigated using non-parametric tests. Spearman’s correlation was used for ordinal variables. The Mann–Whitney test was applied to binary variables and the Kruskal–Wallis test to variables with three or more categories. Effect sizes were calculated as *r* for comparisons between two groups and as *η*^2^ for comparisons between three or more groups. They were classified as negligible (*r* < 0.10), small (0.10 ≤ *r* < 0.30), moderate (0.30 ≤ *r* < 0.50), or large (*r* ≥ 0.50).

To verify whether monthly income and education level influence physical function independently, multiple linear regression models (MLRMs) were fitted. The explanatory variables in all models were monthly income, education level, age, sex, and body mass index (BMI). Given the small sample size, the models were exploratory in nature and did not present multicollinearity problems between the variables.

## 3. Results

### 3.1. Study Population

Of the 73 iwPTLDs evaluated for inclusion in the study, four were excluded due to the presence of neurological/musculoskeletal conditions limiting the execution of the 6MST. Of the 69 iwPTLDs included in the study, 44 (66.8%) were women, with a mean age of 50 ± 15 years. The mean BMI was 25.3 ± 6.3 kg/m^2^, while the median time since the end of treatment for PTB was 16 [4,5,6,7,8,9,10,11,12,13,14,15,16,17,18,19,20,21,22,23] months. The majority were women (63.8%) and of brown color (53.6%). None of the participants had a clinically diagnosed case of extrapulmonary TB. The following findings were described in the evaluation of the chest X-ray: bronchiectasis (*n* = 35, 50.7%), nodular opacities (*n* = 33, 47.8%), pulmonary fibrosis (*n* = 32, 46.4%), pulmonary cavitation (*n* = 16, 23.2%), and others (*n* = 41, 59.4%). The sociodemographic characteristics, comorbidities, and SDoHs of the participants are shown in Table 1.

### 3.2. Functional Exercise Capacity, Muscle Function, and Lung Function

Participants’ performance on the 6MST showed a median of 88 (57–117) steps climbed, corresponding to 50.1% (34.9–73.2%) of the predicted value. Notably, 59 participants (85.5%) performed the test below 80% of the predicted value. Regarding the QMS test, the mean score was 28.7 ± 11.9 kgf. Eleven participants (17.4%) had scores below the cutoff point that characterizes an anormal QMS test result. Spirometry revealed normal, obstructive, restrictive, and mixed patterns in 19 (27.5%), 20 (29%), 18 (26.1%), and 12 (17.4%) of the participants, respectively. The mean FEV_1_ and maximum inspiratory pressure (MIP) values were 68.7% and 57.3% of the predicted values, respectively. In body plethysmography, the mean residual volume (RV)/TLC ratio was 42.7% ± 16%, indicating significant air trapping in this patient population. Data on FEC, muscle function, and pulmonary function are shown in Table 2.

### 3.3. Associations Between Performance on the Six-Minute Step Test and Social Determinants of Health

According to the *p*-value, there was no association between performance on the 6MST and any of the SDoHs. Effect sizes were mostly negligible or small. The associations between 6MST performance and SDoHs are presented in Table 3.

### 3.4. Associations Between Quadriceps Muscle Strength and Social Determinants of Health

The QMS showed a statistically significant association with treated sewage (*W* = 84, *p* = 0.026) and a small effect size (*r* = 0.269). This indicates that participants without access to treated sewage have lower QMS scores. Household economic participation (*H* = 7.788; *p* = 0.051; *η*^2^ = 0.074) and treated water (*W* = 32; *p* = 0.050; *r* = 0.237) were at the threshold of significance, with small effects. The associations between QMS and SDoHs are presented in Table 4.

### 3.5. Associations Between Forced Expiratory Volume in One Second and Social Determinants of Health

FEV_1_ showed a significant positive correlation with education level (*ρ* = 0.248, *p* = 0.040), indicating that participants with higher education levels tend to have better lung function. FEV_1_ was also significantly associated with the presence of social protection after PTB (*W* = 207, *p* = 0.050, *r* = 0.238), suggesting that individuals with worse lung function deterioration were receiving social protection. FEV_1_ was also significantly associated with access to treated water (*W* = 24.5, *p* = 0.029, *r* = 0.264), showing better performance among iwPTLD patients who had access to this government resource. The association between FEV1 and treated sewage was near the threshold of significance (*p* = 0.055), showing a small effect in the same direction. The associations between FEV_1_ and SDoHs are presented in Table 5.

### 3.6. Associations Between Maximum Inspiratory Pressure and Social Determinants of Health

MIP showed a significant positive correlation with education level (*ρ* = 0.246, *p* = 0.042), indicating greater respiratory muscle strength among more educated participants. MIP was also associated with access to treated water (*W* = 20, *p* = 0.021, *r* = 0.280), suggesting weaker respiratory muscle strength among those without access to this resource. Treated sewage was at the threshold of significance (*p* = 0.050) with a small effect in the same direction. The associations between MIP and SDoHs are presented in Table 6.

### 3.7. Associations Between Residual Volume/Total Lung Capacity Ratio and Social Determinants of Health

The RV/TLC ratio showed a stronger and more statistically significant association with the presence of treated sewage at home (*W* = 290, *p* = 0.008, *r* = 0.334) with a moderate effect size. Government social protection after PTB was close to the threshold of significance (*p* = 0.093), with a small effect size. The associations between the RV/TLC ratio (air trapping) and SDoHs are presented in Table 7.

### 3.8. Multiple Linear Regression Model for Performance on the Six-Minute Step Test

The MLRM for performance on the 6MST showed that 12% of the variability in the 6MST is explained by the studied variables. Age was the only variable with a statistically significant association: for each additional year of life, participants climbed an average of 0.7 fewer steps in the test, regardless of other characteristics. There was a positive trend in monthly income: participants with higher income tended to climb more steps. However, this difference was not consistent enough to confirm statistical significance in this sample. Education level had practically no influence on the result. The adjusted MLRM for the 6MST, with the number of steps climbed in the test as the response variable, is presented in Table 8.

### 3.9. Multiple Linear Regression Model for Quadriceps Muscle Strength

The MLRM for the QMS showed that 45% of the variability in the QMS is explained by the studied variables. Male sex was the strongest predictor, with men averaging approximately 13 kgf more than women. Age had a significant negative effect; for each additional year, the QMS decreased by an average of 0.3 kgf, regardless of other variables. BMI also contributed positively. The adjusted MLRM for the QMS is shown in Table 9.

## 4. Discussion

The main findings of the present study were that, in iwPTLD, there is low FEC, impaired lung function, and SDoHs deterioration, even under adequate housing conditions. The association between physical function and SDoHs is weak. The association between lung function (performance in spirometry, respiratory muscle strength, and body plethysmography) and SDoHs is weak to moderate. To our knowledge, this is the first study to evaluate the relationship between SDoHs—which were previously only assessed in PTB—and body functionality (including physical and pulmonary function) in iwPTLD.

Regarding lung function, an obstructive pattern is more common initially in PTLD, while a restrictive pattern becomes more prominent as scarring and fibrosis progress [23]. In our sample, approximately 74% of iwPTLD had never smoked, and 46% of them had obstructive damage (“pure” obstructive or mixed pattern). This finding reinforces the importance of mechanisms other than tobacco in the development of PTLD. Regarding FEC, approximately 85% of our sample performed poorly on the 6MST. In iwPTLD, factors contributing to low FEC include lower ventilatory efficiency and myopathy associated with chronic inflammation, which reduces muscle oxidative capacity and leads to early fatigue, limiting simple tasks such as climbing and descending stairs [1]. Since the 6MST is easy to administer, inexpensive, and highly sensitive for longitudinal monitoring, it can be incorporated into clinical practice to identify functional deficits early and guide personalized interventions [7].

Although exposure to tobacco smoke and environmental pollution in impoverished areas can exacerbate PTLD, the SDoHs associated with PTLD remain understudied [24]. In our study, we found that 71% of iwPTLD had a monthly income of up to one and a half times the minimum wage, indicating a high level of economic vulnerability. Furthermore, only 5.8% of participants reported receiving social benefits after PTLD, which is a low proportion considering the degree of economic vulnerability identified. These findings are concerning and highlight the need to review public policies directed at PTLD. A study conducted in Malawi found that families affected by PTLD remained economically vulnerable 12 months after treatment ended, with more individuals living in poverty (earning USD $1.90/day) than before the PTLD diagnosis [25]. Another study showed that, at the end of treatment, 54% of participants reported that PTB continued to interfere with their work or school participation, 18% reported ongoing disruption in family life, and 9% reported ongoing disruption in social life [26].

In addition to increasing the risk of PTB reinfection, poverty conditions favor malnutrition, as well as overcrowded and poorly ventilated housing and workplaces. These conditions can worsen pulmonary health and lead to serious complications after PTB treatment [13]. Surprisingly, the majority of our iwPTLD sample had access to garbage collection, treated sewage, and treated water, despite the low income. Although the association between sewage treatment (a recognized SDoH) and muscle strength (a determinant of all-cause mortality) may seem like a spurious correlation, a study in Brazil showed that a lack of sewage treatment increased the risk of TB infection 13.5-fold in Rio de Janeiro (*p* < 0.001) [27]. Therefore, SDoHs can indirectly impact health outcomes by serving as indicators of social vulnerability and increased risk of complications in iwPTLD.

In iwPTLD, common CRDs that are often found in the general population can develop, which can lead to worsening pulmonary function. This is because many locations with a high incidence of PTB also have other risk factors for poor pulmonary health, such as malnutrition and indoor and outdoor air pollution [5]. In our study, we demonstrated a weak to moderate relation between pulmonary function and SDoHs. Specifically, basic sanitation conditions, particularly access to treated water and sewage treatment, were the most consistent SDoHs associated with pulmonary function parameters in this sample. Higher educational levels showed significant positive correlations with FEV_1_ and MIP, suggesting that they may be related to better preservation of pulmonary function. The only association with a moderate effect size was between access to treated sewage and RV/TLC, indicating greater air trapping among participants without access to treated sewage. Overall, these findings underscore the importance of monitoring pulmonary function continuously in iwPTLD. Since MIP measurement is inexpensive, simple, and widely available, it could be a useful tool for monitoring iwPTLD in areas with limited resources.

Monitoring of iwPTLD should consider physiological parameters as well as SDoHs contributing to overall functional limitation [28]. In our study, the association between physical function (performance on the 6MST and QMS) and SDoHs was weak although it should not be neglected. In this regard, the study by Choi and colleagues [29] is important because it showed that lower income was associated with an increased risk of long-mortality, while higher BMI and regular physical exercise were associated with a reduced risk of long-term mortality in iwPTLD.

To assess the effect of each variable and to control for the influence of the others, we developed explanatory models for physical function variables. In our sample, biological characteristics (especially sex, age, and BMI) explain much more of the variation in physical function than SDoH. This does not mean that SDoHs are irrelevant to the health of this population but rather that, when present, its effects can operate indirectly. Alongside poverty and air pollution, it is important to note that certain risk factors predispose individuals to PTLD, including older age and low BMI [30]. Furthermore, some SDoHs, such as lower education, low income, and limited family support, can directly influence functional performance, adherence to rehabilitation programs, and social reintegration, thereby amplifying the effects of PTLD on overall health [12,31]. Early detection, clinical assessment, and personalized management of iwPTLD through social protection and pulmonary rehabilitation actions can mitigate functional disability [5].

There are several limitations to the present study that should be noted. First, its cross-sectional design prevents us from establishing cause-and-effect relationships. Therefore, longitudinal studies are essential for determining the impact of SDoHs on the physical and pulmonary functions of iwPTLD. Second, the absence of statistical significance in most comparisons should be interpreted with caution considering the small sample size, which limits statistical power to detect small effects. Although a sample size of 69 participants is adequate for an exploratory investigation, it reduces the ability to detect small-magnitude associations. This is particularly relevant in models with multiple predictors, where statistical power tends to be lower [32]. Third, the lack of detailed measures of respiratory health and socioeconomic conditions at the start of PTB treatment limits our ability to evaluate changes in exercise performance and SDoHs during the post-treatment period. Finally, we did not assess symptoms and QoL; these assessments could have shown stronger correlations with SDoHs in iwPTLD. Despite these limitations, our findings may serve as a basis for randomized clinical trials that implement social protection measures not only during, but also after, PTB treatment to mitigate the impact of socioeconomic conditions on physical performance.

## 5. Conclusions

In iwPTLD, impairments in physical and lung function and deterioration of SDoHs occur. While the relationship between impaired physical function and SDoHs is weak, it should not be overlooked as it can act indirectly. Understanding the relationship between physical function and SDoH can help us develop personalized rehabilitation strategies for iwPTLD that consider their physical limitations and social contexts.

## Figures and Tables

**Table 1:** Sociodemographic characteristics, comorbidities, and social determinants of health in individuals with post-tuberculosis lung disease.

Variables		Values
**Sex (female/male)**		44/25
**Race/color**	Brown	37 (53.6%)
	Black	19 (27.5%)
	White	12 (17.4%)
	Yellow	1 (1.4%)
**COPD**	No	66 (95.7%)
	Yes	3 (4.3%)
**Asthma**	No	67 (97.1%)
	Yes	2 (2.9%)
**Hypertension**	No	47 (68.1%)
	Yes	22 (31.9%)
**Diabetes mellitus**	No	61 (88.4%)
	Yes	8 (11.6%)
**Smoking**	Never smoked	51 (73.9%)
	Former smoker	9 (13%)
	Current smoker	9 (13%)
**Physical activity**	No	43 (62.3%)
	Yes	26 (37.7%)
**Education level**	No education	1 (1.4%)
	Incomplete primary education	15 (21.7%)
	Completed primary education	8 (11.6%)
	Incomplete secondary education	7 (10.1%)
	Completed secondary education	32 (46.4%)
	Incomplete higher education	4 (5.8%)
	Completed higher education	2 (2.9%)
**Monthly income**	None	7 (10.1%)
	Up to 0.5 minimum wage ^1^	2 (2.9%)
	0.6 to 1 minimum wage	18 (26.1%)
	1.1 to 1.5 minimum wage	22 (31.9%)
	1.6 to 2 minimum wages	15 (21.7%)
	2.1 to 5 minimum wages	4 (5.8%)
	>5 minimum wages	1 (1.4%)
**Social protection**	No	65 (95.2%)
	Yes	4 (5.8%)
**Household economic participation**	Does not work	21 (30.4%)
	Responsible for support	18 (26.1%)
	Works but not financially independent	16 (23.2%)
	Financially independent	14 (20.3%)
**Garbage collection**	No	68 (98.6%)
	Yes	1 (1.4%)
**Treated sewage**	No	63 (91.3%)
	Yes	6 (8.7%)
**Treated water**	No	66 (95.7%)
	Yes	3 (4.3%)

The values shown are number (frequency). ^1^ The Brazilian minimum wage is equivalent to approximately 320 United States dollars.

**Table 2:** Functional exercise capacity using the six-minute step test, muscle function using quadriceps and respiratory muscle strength, and lung function using spirometry in individuals with post-tuberculosis lung disease.

Variables		Values
**Functional exercise capacity**	6MST (number of steps climbed)	88 (57–117)
	6MST (% predicted)	50.1 [34.9–73.2]
**Muscle function**	QMS (kgf)	28.7 ± 11.9
	MIP (% predicted)	57.3 ± 24.9
	MEP (% predicted)	57.3 ± 24.9
**Lung function**	FVC (% predicted)	76.7 ± 23.8
	FEV_1_ (% predicted)	68.7 ± 26.6
	FEV_1_/FVC (%)	73.4 ± 15.8
	TLC (% predicted)	85.7 ± 19.6
	RV (% predicted)	96 [81–113]
	RV/TLC (%)	42.7 ± 16

The values shown are the mean ± SD or median (interquartile range). 6MST, six-minute step test; QMS, quadriceps muscle strength; MIP, maximum inspiratory pressure; MEP, maximum expiratory pressure; FVC, forced vital capacity; FEV_1_ = forced expiratory volume in one second; TLC, total lung capacity; RV, residual volume.

**Table 3:** Associations between performance on the six-minute step test and social determinants of health in individuals with post-tuberculosis lung disease.

Social Determinant	Test	Statistic	*p*-Value	Effect Size
**Education level**	Spearman	*ρ* = 0.118	0.336	Small
**Monthly income**	Spearman	*ρ* = 0.181	0.136	Small
**Social protection**	Mann–Whitney	*W* = 127	0.949	*r* = 0.009 (negligible)
**Household economic participation**	Kruskal–Wallis	*H* = 1.779	0.620	*ƞ*^2^ = −0.019
**Garbage collection**	Mann–Whitney	*W* = 20	0.498	*r* = 0.085 (negligible)
**Treated sewage**	Mann–Whitney	*W* = 102	0.065	*r* = 0.223 (small)
**Treated water**	Mann–Whitney	*W* = 44	0.109	*r* = 0.195 (small)

**Table 4:** Associations between quadriceps muscle strength and social determinants of health in individuals with post-tuberculosis lung disease.

Social Determinant	Test	Statistic	*p*-Value	Effect Size
**Education level**	Spearman	*ρ* = 0.162	0.185	Small
**Monthly income**	Spearman	*ρ* = 0.104	0.395	Small
**Social protection**	Mann–Whitney	*W* = 179	0.213	*r* = 0.151 (small)
**Household economic participation**	Kruskal–Wallis	*H* = 7.788	0.051	*ƞ*^2^ = −0.019
**Garbage collection**	Mann–Whitney	*W* = 11	0.258	*r* = 0.139 (small)
**Treated sewage**	Mann–Whitney	*W* = 84	0.026	*r* = 0.269 (small)
**Treated water**	Mann–Whitney	*W* = 32	0.050	*r* = 0.237 (small)

**Table 5:** Associations between forced expiratory volume in one second, as measured by spirometry, and social determinants of health in individuals with post-tuberculosis lung disease.

Social Determinant	Test	Statistic	*p*-Value	Effect Size
**Education level**	Spearman	*ρ* = 0.248	0.040	Small
**Monthly income**	Spearman	*ρ* = 0.115	0.348	Small
**Social protection**	Mann–Whitney	*W* = 207	0.050	*r* = 0.238 (small)
**Household economic participation**	Kruskal–Wallis	*H* = 4.355	0.226	*ƞ*^2^ = −0.021
**Garbage collection**	Mann–Whitney	*W* = 21.5	0.547	*r* = 0.076 (negligible)
**Treated sewage**	Mann–Whitney	*W* = 98.5	0.055	*r* = 0.232 (small)
**Treated water**	Mann–Whitney	*W* = 24.5	0.029	*r* = 0.264 (small)

**Table 6:** Associations between maximum inspiratory pressure and social determinants social of health in individuals with post-tuberculosis lung disease.

Social Determinant	Test	Statistic	*p*-Value	Effect Size
**Education level**	Spearman	*ρ* = 0.246	0.042	Small
**Monthly income**	Spearman	*ρ* = 0.073	0.552	Negligible
**Social protection**	Mann–Whitney	*W* = 187.5	0.143	*r* = 0.178 (small)
**Household economic participation**	Kruskal–Wallis	*H* = 3.464	0.326	*ƞ*^2^ = −0.007
**Garbage collection**	Mann–Whitney	*W* = 22	0.564	*r* = 0.073 (negligible)
**Treated sewage**	Mann–Whitney	*W* = 96.5	0.050	*r* = 0.237 (small)
**Treated water**	Mann–Whitney	*W* = 20	0.021	*r* = 0.280 (small)

**Table 7:** Associations between air trapping, as measured by body plethysmography, and social determinants of health in individuals with post-tuberculosis lung disease.

Social Determinant	Test	Statistic	*p*-Value	Effect Size
**Education level**	Spearman	*ρ* = −0.114	0.370	Small
**Monthly income**	Spearman	*ρ* = −0.011	0.928	Negligible
**Social protection**	Mann–Whitney	*W* = 59	0.093	*r* = 0.211 (small)
**Household economic participation**	Kruskal–Wallis	*H* = 4.356	0.226	*ƞ*^2^ = 0.023
**Garbage collection**	Mann–Whitney	*W* = 58	0.159	*r* = 0.179 (small)
**Treated sewage**	Mann–Whitney	*W* = 290	0.008	*r* = 0.334 (moderate)
**Treated water**	Mann–Whitney	*W* = 116	0.446	*r* = 0.097 (negligible)

**Table 8:** Multiple linear regression model for performance on the six-minute step test in individuals with post-tuberculosis lung disease (R^2^_adjusted_ = 0.121).

Variable	B	95% CI	*p*-Value
**Monthly income**	5.603	−1.462; 12.669	0.118
**Education level**	0.010	−6.619; 6.638	0.998
**Age**	−0.658	−1.260; −0.056	0.033
**Male**	15.404	−3.093; 33.900	0.101
**Body mass index**	−1.064	−2.484; 0.357	0.140

**Table 9:** Multiple linear regression model for quadriceps muscle strength in individuals with post-tuberculosis lung disease (R^2^_adjusted_ = 0.451).

Variable	B	95% CI	*p*-Value
**Monthly income**	0.769	−0.956; 2.493	0.377
**Education level**	0.021	−1.597; 1.639	0.980
**Age**	−0.303	−0.450; −0.156	<0.001
**Male**	12.956	8.441; 17.471	<0.001
**Body mass index**	0.509	0.162; 0.856	0.005

## Data Availability

The original contributions presented in this study are included in the article. Further inquiries can be directed to the corresponding author.

## Abbreviations

The following abbreviations are used in this manuscript:

PTLD	Post-tuberculosis lung disease
TB	Tuberculosis
ADL	Activity of daily living
PTB	Pulmonary tuberculosis
iwPTLD	Individuals with post-tuberculosis lung disease
FEC	Functional exercise capacity
CRD	Chronic respiratory disease
6MST	Six-minute step test
SDoHs	Social determinants of health
QoL	Quality of life
FEV_1_	Forced expiratory volume in one second
FVC	Forced vital capacity
TLC	Total lung capacity
QMS	Quadriceps muscle strength
MLRM	Multiple linear regression model
BMI	Body mass index
MIP	Maximum inspiratory pressure
RV	Residual volume
